# The relationship between fear of COVID-19, psychological well-being and life satisfaction in nursing students: A cross-sectional study

**DOI:** 10.1371/journal.pone.0264970

**Published:** 2022-03-10

**Authors:** Özlem Tekir

**Affiliations:** Faculty of Health Sciences Department of Nursing, Internal Medicine Nursing, İzmir Demokrasi University, İzmir, Turkey; Satyawati College (Eve.), University of Delhi, INDIA

## Abstract

**Objective:**

This study was conducted to examine the relationship between fear of COVID-19, psychological well-being, and satisfaction with life in nursing students.

**Methods:**

A descriptive and cross-sectional design was used in this study. The study was carried out at a university Faculty of Health Sciences Department of Nursing between May 17 and June 25, 2021. The JASP 0.14.1. Software was used for statistical analysis. Kruskal-Wallis test was used for the comparison of three or more groups, Mann-Whitney U test was used for the comparisons of two groups, and Bonferroni-corrected Mann-Whitney U test was used for post hoc analysis. To determine the risk factors for "The Fear of COVID-19 Scale," linear regression analysis with backward stepwise modeling was used.

**Results:**

The mean score of the students was 18.48±6.87 from the Fear of COVID-19 Scale, 38.42±12.60 from the Psychological Well-Being Scale, and 13.12±4.97 from the Satisfaction with Life Scale. According to the results of the regression model established, independent variables explained 12.5% of the dependent variables, but the regression model established was found to be statistically significant. A one-unit increase in the satisfaction with life scale increased the fear of COVID-19 scale score by 0.224 units, and this increase was found statistically significant (p = 0.030) as a result of linear regression analysis used with backward stepwise modeling.

**Conclusion:**

In conclusion, it was found that the students’ fear of COVID-19 was below the medium level, their psychological well-being was above the medium level, and that their life satisfaction was below the medium level.

## Introduction

The COVID-19 epidemic first appeared with the emergence of an unknown pneumonia outbreak in Wuhan, the Hubei province of China on 31 December 2019 with a new coronavirus (2019-nCoV) and spread rapidly around the world, eventually leading to a global pandemic. Because of the similarity between the emerging new virus and SARS-CoV, it was called SARS-CoV-2 [1, 2]. This infection was named COVID-19 by the WHO on February 11, 2020 and declared a pandemic on March 11, 2020 [3]. The first case appeared in Turkey on March 11, 2020 [4].

Life satisfaction means how much the individual enjoys their life [5] and includes cognitive judgments about personal life as one of the complements of happiness and subjective well-being [6]. The indicators of satisfaction with life have been determined as physical, social, emotional, mental health, psychological well-being, having functional and effective communication skills, initiating and maintaining social relationships, and having social connections [7]. One of the segments of society where satisfaction with life is important and which reflect the perceptions and evaluations of the individual about their life include university students. University life is a period in which students experience their adult roles, prepare for working life, and idealize the values related to their lives more [8].

The concept of well-being is one of the important concepts of positive psychology [9]. Subjective well-being can be related to living well. Similarly, living well can involve a life experience that is and has been lived well, which means satisfaction with life [10]. Psychological well-being involves individuals’ positive perception of themselves and awareness of their strengths and limitations by recognizing themselves realistically, being satisfied with themselves, being able to act autonomously and independently, and finding their life meaningful [11]. In a study, psychological well-being was found to be negatively related to disorders, such as depression, anxiety, and temper [12]. Also, the relationship between the concept of psychological well-being and social anxiety has been studied, and it has been reported that social anxiety impairs functionality in social relationships, causes difficulties in interpersonal relationships, and thus leads to a decrease in psychological well-being [13]. Individuals with low psychological well-being make negative evaluations of their lives more frequently [14]. In addition, in another study on satisfaction with life and psychological well-being, it was emphasized that low autonomy and low social relationships and deterioration in social relationships caused a significant decrease in satisfaction with life and psychological well-being [15].

Evaluations, such as the emergence of the pandemic and disruption of the routine life, feeling of uncertainty, fear of getting infected, and thinking that the environment is unsafe, have shown that the pandemic has psychological effects as well as physiological ones [16–18]. Fear is defined as an unpleasant emotional state triggered by the perception of threatening stimuli. Extraordinary situations such as pandemics can arouse fear in many people [19]. The concept of the fear of COVID-19, on the other hand, refers to people’s anxious and fearful mood resulting from their fear of getting infected with COVID-19 [20, 21]. The anxiety experienced in the pandemic is not limited to individuals themselves. They also feel anxious about their family and loved ones, sometimes even more than themselves, and they may experience intense emotions, such as anxiety, panic, and fear. Being at risk for contracting the disease, not knowing exactly when the pandemic will end, uncertainty about the social and economic difficulties that may be experienced during the pandemic process, and most importantly, our concerns about how to protect ourselves and our family in this process can inevitably cause us to experience intense stress and anxiety [22].

Fear is a multifaceted factor, and it is frequently one of the most significant underlying factors of compromised mental health and well-being [23]. If fear reaches an uncontrollable level, it may threaten the mental health of individuals. Mental health is an issue that interests the general well-being and psychological well-being of both individuals and society [24]. According to the literature, the fear of COVID-19 has a negative impact on individuals’ satisfaction with life, too [25, 26]. At this very point, the COVID-19 pandemic process leads to pessimistic feelings and thoughts in adolescents [27]. It is thought that coping with these feelings and thoughts will positively affect the level of adolescents’ well-being.

Today, there is no detailed information on the effects of the COVID-19 pandemic on people’s mental health both in the world and in our country [28]. This situation also obscures what psychosocial services (psychosocial support programs carried out by professionals, information services, online counseling, training programs and activities that include coping strategies and increase resilience, psychological counseling centers on campuses) may be offered to the target population both during and after the epidemic. For this reason, it is thought that it is important to make psychological assessments of undergraduate students at a time when the effects of the pandemic still dominate in our country [29]. Within the scope of the research, participants’ satisfaction with life, which is one of the indicators of the level of well-being, was also evaluated.

As far as I know, this is the first study in Turkey to examine the relationship between fear of COVID-19, psychological well-being, and satisfaction with life by using respective scales.

Reflecting on these concepts, which are considered to be associated with each other, this study was conducted to investigate the relationship between the fear of COVID-19 and psychological well-being and satisfaction with life in nursing students.

### Research questions

1. What is the level of nursing students’ fear in the COVID-19 process and what are the factors affecting it?
2. What is the level of nursing students’ psychological well-being in the COVID-19 process and what are the factors affecting it?
3. What is the level of nursing students’ satisfaction with life in the COVID-19 process and what are the factors affecting it?
4. What is the relationship between the level of nursing students’ fear of COVID-19 and psychological well-being and satisfaction with life?

## Materials and methods

### Design and sample

A descriptive and cross-sectional design was used in this study. The study was carried out in the Izmir Democracy University Faculty of Health Sciences Department of Nursing between May 17 and June 25, 2021. The population of the study consisted of first, second, and third-year nursing students (N = 220). Since the population of the study also constituted the sample, no sample limitation was applied. The sample of the study consisted of students who volunteered to participate in the study (n = 171).

### Data collection tools

The study data were collected via an online questionnaire form that included a Personal Information Form, the Fear of COVID-19 Scale, the Psychological Well-Being Scale, and the Satisfaction with Life Scale. To do this, the authors created an online questionnaire specifically for this research on Google forms.

#### The personal information form

The personal information form consisted of a total of 11 questions about students’ age, gender, school year, the high school that was graduated, place of residence, level of mother’s education, level of father’s education, family income, mother’s working status, father’s working status, and family type.

#### The fear of COVID-19 scale

This 7-item scale was developed by Ahorsu et al. (2020), and its adaptation into Turkish and its validity and reliability study were conducted by Satıcı et al. (2020). The target age range of the scale is wide and can be used on university students and adults. All of the items of the scale are scored positively. The scale uses a 5-point Likert-type scoring system with options ranging from 1 to 5 (1- strongly disagree; 5-strongly agree). There are no reverse-scored items. Scores range between 7 and 35. A high score indicates a high level of fear of the COVID pandemic. In the Turkish validity and reliability study of the scale, Cronbach’s Alpha value was found to be α = .82. In this study, the alpha value was determined as α = .90 [20, 25].

#### The psychological well-being scale

This scale was developed by Diener et al. (2009–2010) to measure socio-psychological well-being, which is complementary to existing well-being measures. The Turkish adaptation of the scale was conducted by Telef (2011; 2013). Cronbach’s alpha internal consistency coefficient obtained in the reliability study of the scale was calculated as .80. The items on the scale are responded with options ranging between “I strongly disagree (1)” and “I strongly agree (7)”. All items are expressed positively. Scores range from 8 (strongly disagree to all items) to 56 (strongly agree to all items). A high score indicates that the person has many psychological resources and strengths [30, 31].

#### The satisfaction with life scale

This scale was developed by Diener, Emmons, Laresen, and Griffin (1985) and was adapted into Turkish by Köker (1991). It consists of five items related to satisfaction with life. Each item is evaluated on a 7-point Likert-type scale (1: not at all appropriate—7: very appropriate). The scale, which aims to measure general satisfaction with life, is suitable for all ages, from adolescents to adults. The translation of the scale into Turkish and its validity study with the "face validity" technique were carried out by Köker (1991). As a result of the item analysis, the correlation between the score of each item on the scale and the total score was found to be adequate. The test-retest reliability coefficient of the scale was determined as .85. A second adaptation of the scale from English into Turkish was conducted by Dağlı and Baysal (2016). They reduced the evaluation options to five as follows: 1- I completely disagree; 2- I slightly agree; 3- I moderately agree; 4- I agree to a great extent; and 5- I completely agree [32]. The lowest score that can be obtained from the current scale is 5, and the highest score is 25. A low score indicates low satisfaction with life.

The results of the reliability analysis of the scales were examined in this study, too. The reliability of the three scales in the study was found to be quite high. The coefficients of the reliability analysis (Cronbach’s Alpha values) were found to be 0.903 for the Fear of COVID-19 Scale, 0.942 for the Psychological Well-Being Scale, and 0.906 for the Satisfaction with Life Scale.

### Statistical analysis

The JASP 0.14.1. Software was used for statistical analyses. Descriptive statistics were presented as frequency and percentage values for categorical variables, and as mean, standard deviation, median, and minimum and maximum values for numeric variables. Shapiro-Wilk test was used for the test of normality. Kruskal-Wallis test was used for the comparison of 3 or more groups, Mann-Whitney U test was used for the comparison of 2 groups, and Bonferroni-corrected Mann-Whitney U test was used for post hoc analysis. Spearman correlation test was used for evaluating the relationship between numeric variables. To determine the risk factors for "The Fear of COVID-19 Scale," linear regression analysis with backward stepwise modeling was used. Cronbach’s alpha coefficients were calculated for each scale used in the study for reliability. For all statistical comparisons, a p-value below 0.05 was assumed as statistically significant.

### Ethical considerations

Ethics committee approval of the Ministry of Health General Directorate of Health Services Scientific Research Platform and the Clinical Research Ethics Committee was obtained for the implementation of the study (Decision no:2021/05-10; Date: 28/04/2021). The permission of the researchers who conducted the Turkish validity and reliability study of the Fear of COVID-19 Scale, the Psychological Well-Being Scale, and the Satisfaction with Life Scale was obtained. The research data were collected from students via an online questionnaire. Students checked a box on the questionnaire to state their voluntary participation in the study before starting to answer the questions.

## Results

### Participant characteristics

Of the students, 69.0% were female, 32.7% were 20 years old, 49.1% were 1st-year students, 25.1% were 2nd-year students, 25.7% were 3rd-year students, 76.6% were Anatolian high school graduates, 33.3% were living in metropolitan cities, and 77.2% had a nuclear family (Table 1).

**Table 1:** Socio-demographic characteristics of the students.

Sociodemographic Characteristics		n	%
**Age**	18	14	8,2
	19	32	18,7
	20	56	32,7
	21	46	26,9
	22+	23	13,5
**Gender**	Female	118	69,0
	Male	53	31,0
**School year**	1^st^ -year	84	49,1
	2^nd^-year	43	25,1
	3^rd^-year	44	25,7
**Graduation**	Normal high school	5	2,9
	Anatolian high school	131	76,6
	Science high school	25	14,6
	Health vocational high school	10	5,8
**The longest place of residence**	Village	23	13,5
	County	51	29,8
	Province	40	23,4
	Metropolis	57	33,3
**Mother’s education**	Not literate	30	17,5
	Literate	14	8,2
	Primary education	88	51,5
	High school	28	16,4
	Associate/Undergraduate degree	11	6,4
**Father’s education**	Not literate	9	5,3
	Literate	13	7,6
	Primary education	88	51,5
	High school	38	22,2
	Associate/Undergraduate degree	20	11,7
	Graduate degree	3	1,8
**Family income**	Income<expenses	34	19,9
	Income = expenses	115	67,3
	Income>expenses	22	12,9
**Employment status of the mother**	Housewife	128	74,9
	Retired	11	6,4
	Working	32	18,7
**Employment status of the father**	Retired	42	24,6
	Working	108	63,2
	Not working	21	12,3
**Type of family**	Immediate family	132	77,2
	Extended family	36	21,1
	Lives alone	3	1,8
**Total**		171	100

### Students’ mean scores from the fear of COVID-19, psychological well-being and satisfaction with life scales

The mean scores of the students were 18.48 ± 6.87 from the Fear of COVID-19 Scale, 38.42 ± 12.60 from the Psychological Well-Being Scale, and 13.12 ± 4.97 from the Satisfaction with Life Scale (Table 2).

**Table 2:** Mean scores on the fear of COVID-19, psychological well-being, and satisfaction with life scales.

	Mean ± SD	Median	Min.–Max.
**Fear of COVID-19 Scale**	18,48 ± 6,87	18	7–35
**Psychological Well-Being Scale**	38,42 ± 12,60	41	8–56
**Satisfaction with Life Scale**	13,12 ± 4,97	13	5–25

### Comparison of students’ mean scores from the fear of COVID-19, psychological well-being, and satisfaction with life scales according to some socio-demographic characteristics

There was a statistically significant difference between the mean scores of the students from the Fear of COVID-19 Scale by gender (p = 0.001), and the score of females was higher than that of males. There was no statistically significant difference between students’ scores from the Psychological Well-Being Scale by gender (p = 0.463). A statistically significant difference was found between students’ scores from the Satisfaction with Life Scale by gender (p = 0.005), and the score of females was higher than that of males.

There was a statistically significant difference between students’ scores from the Fear of COVID-19 Scale by school year (p = 0.015). There was a statistically significant difference between the scores of the 1st and 2nd-year students (p = 0.005). A statistically significant difference was found between students’ scores from the Psychological Well-Being Scale by school year (p = 0.005). The difference was between the scores of the 1st and 3rd-year students (p = 0.004). There was no statistically significant difference between students’ scores from the Satisfaction with Life Scale by school year (p = 0.172) (Table 3).

**Table 3:** Comparison of the students’ mean scores on the fear of COVID-19, psychological well-being and satisfaction with life scales according to some socio-demographic characteristics.

Characteristics		Fear of COVID-19 Scale	Psychological Well-Being Scale	Satisfaction with Life Scale
**Gender**	Female	19,64 ± 6,75	38,84 ± 12,77	13,82 ± 5,07
	Male	15,91 ± 6,49	37,47 ± 12,26	11,55 ± 4,39
	**Test statistics**	**0,001**	**0,463**	**0,005**
**School year**	1^st^ -year	16,89 ± 5,89	36,15 ± 12,18	12,61 ± 4,44
	2^nd^-year	21,09 ± 7,90	37,56 ± 14,79	12,93 ± 5,97
	3^rd^-year	18,95 ± 6,83	43,57 ± 9,46	14,27 ± 4,80
	**Test statistics**	**0,015**	**0,005**	**0,172**

### The relationship between the fear of COVID-19, psychological well-being, and satisfaction with life scales

There was no statistically significant relationship between the Fear of COVID-19 Scale and the Psychological Well-Being Scale (p = 0.053). A very weak negative correlation was found between the Fear of COVID-19 Scale and the Satisfaction with Life Scale (p = 0.014; r = -0.187). There was a strong positive correlation between the Satisfaction with Life Scale and the Psychological Well-Being Scale (p<0.001; r = 0.710) (Table 4).

**Table 4:** The relationship between the fear of COVID-19, psychological well-being and satisfaction with life scales.

	Fear of COVID-19 Scale	Psychological Well-Being Scale	Satisfaction with Life Scale
**Fear of COVID-19 Scale**	-		
**Fear of COVID-19 Scale**	r = 0,148	-	
	p = 0,053		
**Fear of COVID-19 Scale**	r = -0,187	r = 0,710	-
	p = 0,014	p<0,001	

# Spearman correlation test

### Determination of the risk factors for the fear of COVID-19 scale with linear regression analysis

In addition to the existing statistical analyses in this study, the Fear of COVID-19 Scale, the Psychological Well-Being Scale, and the Satisfaction with Life Scale were analyzed to find out whether they were affected by demographic variables and, if any, what the extent of the effect was. Accordingly, the Fear of COVID-19 was taken as the dependent variable and the Psychological Well-being Scale, the Satisfaction with Life Scale, gender, school year, income, and the level of father’s education were taken as independent variables. According to the results of the regression model established, independent variables explained 12.5% of the dependent variables, but the regression model established was found to be statistically significant (Table 5).

**Table 5:** Determination risk factors for the fear of COVID-19 scale with linear regression analysis.

	Sum of Squares	df	Mean Square	F	p
**Regression**	1002,682	3	42,024	7,953	<0.001
**Residual**	7017,997	167			
**Total**	8020,678	170			

* ANOVA table for the significance of the model

### Determination of the risk factors for the fear of COVID-19 with linear regression analysis used with backward stepwise modeling

The variables that were found to be significant in the model with the backward regression modeling were satisfaction with life scale, gender, and school year. A one-unit increase in the satisfaction with life scale increased the fear of COVID-19 scale score by 0.224 units, and this increase was statistically significant (p = 0.030). The female gender increased the Fear of COVID-19 Scale score by 2.845 units compared to the male gender, and this increase was statistically significant (p = 0.011), too. In other words, it can be said that females experienced higher fear of COVID-19 than males. Being a 2nd-year student also showed a significant increase compared to being a 1st and 3rd-year student (p = 0.007). Being a 2nd-year student increased the Fear of COVID-19 Scale score by 3.165 units. Other variables did not have a significant effect on the dependent variable (Table 6).

**Table 6:** Determination of risk factors for the fear of COVID-19 with linear regression analysis used with backward stepwise modeling.

	B	Std. Error	Std. Beta	t	p	%95 CI Lower—Upper
**Constant**	12,779	1,501		8,513	<0,001	
**YDO**	0,224	0,102	0,162	2,189	0,030	0,953–1,050
**Gender**	2,845	1,107	0,192	2,571	0,011	0,938–1,066
**School year (2)**	3,165	1,153	0,200	2,744	0,007	0,982–1,019

## Discussion

This study was conducted to evaluate the fear of COVID-19, psychological well-being, and satisfaction with life in nursing students and to examine the relationship between the fear of COVID-19, psychological well-being, and satisfaction with life. Health sciences students form a group that represents society, and they are going to be health professionals who will have responsibilities such as determining the needs of society and planning health services in the future; therefore, it is very important to identify the impact of COVID-19 and to find solutions for the problems emerging due to it. In addition, it is thought that one of the vulnerable groups psychologically affected during the pandemic will be university students. Staying away from people they love, possible difficulties they may experience in finding a job in the future, and the impact of the virus on their education life may increase their anxiety levels [28, 33]. One of the first studies conducted on university students during the COVID-19 pandemic indicated that this group showed more signs of anxiety than other segments of society [34].

In this study, there was a statistically significant difference between students’ scores from the Fear of COVID-19 Scale by gender, and the scores of females were higher than those of males. Women are emotionally more sensitive and experience emotions more intensely, which can be considered as the reason for this difference. Similarly, some studies have shown that females’ fear of coronavirus is higher than that of males [16, 24]. For example, Majed et al. (2020) and Broche-Pérez et al. (2020) found that women had a higher level of Coronaphobia than men [35, 36]. Unlike these findings, Aksoy and Atılgan (2021) found that women’s fear of COVID-19 was significantly lower than that of men according to their scores from the Fear of COVID-19 Scale [37]. In their study on students, Cao et al. (2020) found that the psychological effect of the COVID-19 pandemic did not differ according to gender, and that male and female students experienced similar stress and negative emotions due to the pandemic [38]. In some other studies, too, it was stated that there was no significant difference between gender and the fear of COVID-19 [20, 39].

In the current study, there was no statistically significant difference between students’ scores from the Psychological Well-Being Scale by gender. Similar to the finding of this study, Kermen et al. (2016) found that the psychological well-being levels of high school students did not differ by gender [40]. Unlike this study, Geçgin and Sahranç (2017) found that female students had higher total scores on the Psychological Well-being Scale than male students [41]. Erturk et al. (2016) found that the level of psychological well-being differed by gender and that the psychological well-being levels of females were higher than those of males [42]. In a study conducted by Kuyumcu (2012) on Turkish and British university students, it was stated that the psychological well-being level of females was higher than that of males [43]. Ergül-Topçu et al. stated in their study that being female was associated with low psychological well-being [44].

There was a statistically significant difference between students’ scores from the Satisfaction with Life Scale by gender, and the scores of females were found to be higher than those of males. Similarly, Tuzgöl Dost (2007) found significant differences between the levels of students’ satisfaction with life by gender [45]. Yılmaz and Aslan (2013) found that the level of male teachers’ satisfaction with life was significantly lower than that of female teachers [46]. Unlike this study, Aksoy and Atılgan (2021) reported that there was no statistically significant difference between participants’ scores from the satisfaction with life scale by gender variable [37]. It can be thought that the differences between the studies may be due to the sample groups and their size.

In this study, the mean score of the students on the Fear of COVID-19 Scale was 18.48 ± 6.87, and their fear of COVID-19 was below the moderate level. Unlike the findings of this study, Gencer (2020) showed that the group with the youngest mean age was the group with the highest fear of coronavirus. In other words, they stated that those who were afraid of the Coronavirus most were young people aged between 15 and 20 [24]. In their study on 231 healthcare professionals, Mora-Magana et al. (2020) found that 30.3% of the participants were Coronaphobic [47]. Duman (2020) showed that college students’ fear of the COVID-19 pandemic was moderate [48].Çiftçi and Demir (2020) stated that the COVID-19-related fear and stress level of football players was moderate [19]. It can be thought that differences between the studies stem from sample groups and their size. As people get older, the likelihood of contracting various diseases increases, and individuals over the age of 65 and people with concomitant chronic diseases are at risk for contracting coronavirus most [4, 49]. Accordingly, it is an expected result that the level of fear of the disease increases with the advanced age. In this study, the mean score of the students on the Psychological Well-being Scale was 38.42 ± 12.60, and the level of psychological well-being was above the moderate level. Participants’ mean score on the Psychological Well-being Scale was 41.30 in Demir et al. (2021), 38.69± 1.04 in Kermen et al. (2016), and 33.83 in Ergül-Topçu et al.(2021) [14, 40, 44]. In this study, students’ mean score on the Satisfaction with Life Scale was 13.12±4.97, and the level of satisfaction with life was below the moderate level. Participants’ mean score on the Satisfaction with Life Scale was 19.3614 in Demir et al. (2021), 21.48±7.6340 in Kermen et al. (2016), 21.750 in Gündoğar et al. (2007), and 22.49±6.251 in Tekir et al. (2016) [14, 40, 50, 51]. The difference between the findings of the studies may be associated with the differences in the quality and quantity of the settings and samples.

The global pandemic is one of the factors affecting psychological well-being [44]. In this study, there was no statistically significant relationship between the Fear of COVID-19 Scale and the Psychological Well-Being Scale. Similar to this study, Çiçek and Almalı (2020) in their study on anxiety, self-efficacy, and psychological well-being during the COVID-19 pandemic stated that the negative effect of anxiety about the pandemic on psychological well-being was negligible in the public [52]. Anxiety is expressed as an adaptive mechanism for coping with danger, a basic human emotion, and a multifaceted emotional state [53]. The state of fear and anxiety that occurs during the pandemic period is understandable because no one wants to be infected with the virus, but when the fear reaches an uncontrollable state, it leads to consequences that threaten the mental health of individuals [54]. In this study, the mean score from the fear of COVID-19 scale was 18.48 ± 6.87, and the level of fear of COVID-19 was found to be below the moderate level. From another point of view, age has been a known risk factor since the beginning of the pandemic. Various studies in the literature indicate that elderly individuals are at a bigger risk during the pandemic process [55]. In their study on the fear of coronavirus (COVID-19) in elderly individuals, Arısoy and Çay (2021) found that there was a moderate and significant positive relationship between the age of the participants and the fear of Coronavirus and stated that the fear of coronavirus increased as the age increased [55]. From another point of view, the students in the research group were at home with their families at the time of the research. It can be thought that family/social support has a positive effect on the level of psychological well-being. In their study on the anxiety and psychological well-being of individuals during the COVID-19 pandemic, Ateş (2021) found that there was a high, positive, statistically significant, and linear relationship between the Psychological well-being score and the Family Sub-Dimension (r = 0.924; p < 0.001) score [56]. In light of all this information, the reasons why there was no statistically significant relationship between the level of the fear of COVID-19 and Psychological Well-being in this study can be listed as follows: the sample group consisted of young individuals; the level of the fear of COVID-19 of the group was below the medium level; and the students were at home with their families at the time of the research, which provided family support.

Ergül-Topçu (2021) stated that as the level of COVID-19 anxiety increased, psychological well-being decreased [44]. Galasso et al. (2020) stated that females were more aware of the seriousness of the global pandemic. In this case, females felt more threatened and reported less psychological well-being than men. However, they stated that although this situation negatively affected psychological well-being, it caused females to be more careful in taking precautions regarding the global pandemic [57].

Living standards affect individuals positively or negatively in various dimensions, such as psychological, economic, social, educational opportunities, or lack of educational opportunities. At the same time, individuals’ satisfaction with life is affected by these factors. People inevitably experience fear of Coronavirus in more or less different dimensions. In this sense, the COVID-19 pandemic can also significantly affect individuals’ satisfaction with life [37]. In this study, a very weak negative relationship was found between the Fear of COVID-19 Scale and the Satisfaction with Life Scale. However, Aksoy and Atılgan (2021) found no significant relationship between participants’ scores from the Fear of COVID-19 Scale and their satisfaction with life [37]. Similar to this study, they showed the negative effect of anxiety on satisfaction with life during the COVID-19 process [58–60]. A study revealed that the fear of COVID-19 predicted life satisfaction negatively and that depression, anxiety, and stress variables had a mediating role in the relationship between fear of COVID-19 and life satisfaction [25]. In a study conducted in Poland, it was found that the levels of participants’ hope, life satisfaction, and meaning in life negatively predicted their COVID-19 stress levels. In other words, they stated that as the levels of participants’ hope, life satisfaction, and meaning in life increased, their levels of COVID-19 stress decreased [61].

Satisfaction with life has a central role in the lives of young people. It is very important in the development of young people, discovering their talents, coping with stress, and reaching their goals [62]. The determinants of life satisfaction were determined as physical, social, emotional, and mental health, psychological well-being, having the ability to communicate functionally and effectively, initiating and maintaining social relationships, and having social connections [40]. As predicted, there was a strong positive relationship between the Satisfaction with Life Scale and the Psychological Well-being Scale. Demir et al. (2021), too, found a positive and moderate relationship between satisfaction with life and psychological well-being [14].

## Conclusions

In this study, participants’ mean score on the Fear of COVID-19 Scale was 18.48 ± 6.87, and the levels of the fear of COVID-19 were below the moderate level. Also, the mean score of the participants on the Psychological Well-being Scale was 38.42 ± 12.60, and the level of psychological well-being was above the moderate level. The mean score obtained from the Satisfaction with Life Scale was 13.12 ± 4.97, and there was no statistically significant relationship between the COVID-19 Fear Scale and the Psychological Well-Being Scale. On the other hand, there was a very weak negative relationship between the Fear of COVID-19 Scale and the Satisfaction with Life Scale, and a strong positive relationship was found between the Satisfaction with Life Scale and the Psychological Well-Being Scale. The results of the present study can be used to make some recommendations.

The students who made up the study group started to get their COVID-19 vaccines at the end of June 2021, when the study ended. At the time of the research, the students were taking distance education at home. Also, they could not continue their internship education in health institutions/hospitals on these dates and did not work with infected patients with COVID-19. This can be considered as the reason why students’ fear of COVID-19 was below the medium level and their psychological well-being level was above the medium level.

The study reveals that more comprehensive research into this subject is necessary. Since nursing students both form a group that is part of society and represent future health professionals who will have responsibilities such as determining the needs of society and planning health services, it is necessary to determine the impact of COVID-19 and to find solutions to eliminate its effects. In addition, determination of the factors that cause students to experience fear and anxiety in the COVID-19 process is very important to develop methods that will help them to cope with the fear of COVID-19 and to increase their psychological well-being and satisfaction with life in this process.
